# Duration of salmeterol-induced bronchodilation in mechanically ventilated chronic obstructive pulmonary disease patients: a prospective clinical study

**DOI:** 10.1186/cc7117

**Published:** 2008-11-14

**Authors:** Polychronis Malliotakis, Manolis Linardakis, George Gavriilidis, Dimitris Georgopoulos

**Affiliations:** 1 Intensive Care Medicine Department, University Hospital of Heraklion, University of Crete, PO Box 1352, 71110 Heraklion, Crete, Greece

## Abstract

**Introduction:**

Delivery of bronchodilators with a metered-dose inhaler (MDI) and a spacer device in mechanically ventilated patients has become a widespread practice. However, except for the short-acting β2-agonist salbutamol, the duration of action of other bronchodilators, including long-acting β2-agonists, delivered with this technique is not well established. The purpose of this study was to examine the duration of bronchodilation induced by the long-acting β2-agonist salmeterol administered with an MDI and a spacer in a group of mechanically ventilated patients with exacerbation of chronic obstructive pulmonary disease (COPD).

**Methods:**

Ten mechanically ventilated patients with acute exacerbation of COPD received four puffs of salmeterol (25 μg/puff). Salmeterol was administered with an MDI adapted to the inspiratory limb of the ventilator circuit using an aerosol cloud enhance spacer. Static and dynamic airway pressures, minimum (R_int_) and maximum (Rrs) inspiratory resistance, and the difference between Rrs and R_int _(ΔR) were measured before and at 15, 30, and 60 minutes as well as at 2, 3, 4, 6, 8, 10, and 12 hours after salmeterol administration. The overall effects of salmeterol on respiratory system mechanics and heart rate during the 12-hour study period were analyzed by nonparametric Wilcoxon signed rank test.

**Results:**

Salmeterol caused a significant decrease in dynamic and static airway pressures, R_int_, and Rrs. These changes were evident at 30 minutes and remained significant for 8 hours after salmeterol administration. The duration of bronchodilation varied significantly among patients, lasting in some patients more than 10 hours and wearing off in others in less than 6 hours.

**Conclusions:**

It is concluded that four puffs of salmeterol delivered with an MDI and a spacer device induces significant bronchodilation in mechanically ventilated patients with COPD exacerbation, the duration of which is highly variable, precluding definite conclusions in regard to optimum dosing schedules.

## Introduction

Delivery of bronchodilators with metered-dose inhalers (MDIs) in mechanically ventilated patients has become a widespread practice in recent years [1-6]. Several studies have shown that MDIs adapted to the inspiratory limb of the ventilator using a compatible spacer device are as effective as nebulizers despite a significantly lower dose of bronchodilator administered with this technique [1-6]. Compared with the nebulizer, an MDI has several advantages, such as reliability of dosing, ease of administration, reduced cost, less personnel time needed, and lower risk of contamination [7-10]. Although the technique of administration as well as the dose-response curve of bronchodilators in mechanically ventilated patients using an MDI and a spacer device has been examined by several studies [1-3,11-15], data in regard to the duration of the bronchodilator response are scarce and restricted to salbutamol [16,17]. Furthermore, although the bronchodilator response of long-acting β2-agonists – which are considered first-line treatment in symptomatic chronic obstructive pulmonary disease (COPD) [18,19] – has been studied in stable spontaneously breathing COPD patients [20-23], no data exist concerning the effect of long-acting β2-agonists in mechanically ventilated patients with COPD exacerbation. The purpose of the present study, therefore, was to examine the duration of bronchodilation induced by the long-acting β2-agonist salmeterol administered with an MDI and a spacer in a group of mechanically ventilated patients with COPD.

## Materials and methods

Ten male patients (mean age ± standard deviation of 67.8 ± 6 years) with COPD, requiring endotracheal intubation and mechanical ventilation to manage acute respiratory failure due to an acute exacerbation of chronic airflow obstruction, were studied. Patients with a diagnosis of bronchial asthma as well as patients with pneumonia, pulmonary edema, refractory hypoxemia, pneumothorax, or excessive airway secretions were excluded. All patients had a previous diagnosis of COPD and met established criteria for this diagnosis [18]. Their previous pulmonary function data were consistent with severe COPD, with forced expiratory volume in the first second (FEV_1_) of 38% ± 4%, forced vital capacity of 56% ± 6%, residual volume of 177% ± 25%, and total lung capacity of 112% ± 7% of predicted values (mean ± standard error). The study was approved by the hospital ethics committee, and informed consent was obtained from the patients' next of kin. In cases in which patients regained the capacity to give consent, this was later obtained from them.

The patients were studied during a period of clinical stability, no more than 72 hours after the onset of mechanical ventilation. All patients were orotracheally intubated (low-pressure cuff endotracheal tube, internal diameter of 8.0 ± 0.5 mm, and tube length of 28 ± 1 mm), heavily sedated (propofol at a rate of 4 mg/kg per hour and remifentanyl), and ventilated on volume-controlled mode (Evita 2; Draeger, Luebeck, Germany) using settings that minimized dynamic hyperinflation (tidal volume [VT] of 7 to 8 mL/kg, square wave flow-time profile, no end-inspiratory pause, and zero external positive end-expiratory pressure [PEEPe]) and a fractional concentration of inspired oxygen that achieved a hemoglobin saturation of greater than 89%. Minute ventilation was adjusted in each individual by the attending physician in order to maintain normal arterial pH and remained constant throughout the study. The absence of respiratory muscle activity was based on specific criteria, including absence of negative deflection of airway pressure (Paw), stabilization of the Paw waveform, constancy of peak inspiratory pressure from breath to breath, and exponential decline of expiratory flow [24]. Patient characteristics and baseline ventilator settings are shown in Table 1.

**Table 1:** Patient characteristics and baseline ventilator settings

Age, years	FiO_2_	PaO_2_, mm Hg	PaCO_2_, mm Hg	VT, liters	Fr, breaths per minute	V'_I_, liters per second	T_I_/T_TOT_	V_E_, liters per minute
67.8 ± 6.0	0.39 ± 0.06	72.8 ± 7.1	59.4 ± 3.8	0.52 ± 0.04	14.6 ± 1.6	0.72 ± 0.04	0.24 ± 0.02	7.5 ± 0.9

Data are presented as mean ± standard deviation. FiO_2_, fractional concentration of inspired oxygen; Fr, respiratory frequency; PaCO_2_, partial pressure of arterial carbon dioxide; PaO_2_, partial pressure of arterial oxygen; T_I_/T_TOT_, duty cycle; V_E_, minute ventilation; V'_I_, constant inspiratory flow; VT, tidal volume.

Flow at the airway opening was measured with a heated pneumotachograph (Hans-Rudolf 3700; Hans Rudolph, Inc., Shawnee, KS, USA) and a differential pressure transducer (Micro-Switch 140PC; Honeywell Sensing and Control, Golden Valley, MN, USA) placed between the endotracheal tube and the Y-piece of the ventilator circuit. Flow was electronically integrated to provide volume. Paws (Micro-Switch 140PC) were measured from a side port between the pneumotachograph and the endotracheal tube. All signals were sampled at 50 Hz (Windaq, Datac Instruments Inc., Akron, OH, USA) and stored on a computer disk for later analysis.

Each patient received four puffs of salmeterol. Each puff contained 25 μg of salmeterol xinafoate and was given by an MDI canister (Serevent inhaler; Glaxo Smith Kline, Uxbridge, Middlesex, UK) adapted to the inspiratory limb of the ventilator circuit using an aerosol cloud enhancer spacer (Diemolding Healthcare Division, Canastota, NY, USA), whereby the MDI flume is directed away from the patient. The spacer was placed just before the Y-ventilator connector. The canister was shaken before each series of puffs. Each puff was delivered at 20- to 30-second intervals, immediately before initiation of airflow by the ventilator. Inspiratory flow and VT during administration were kept constant to baseline values (Table 1). All bronchodilators were withheld at least 6 hours before the study. All patients were receiving corticosteroids (1 mg/kg of body weight intravenous prednisolone per day) and this regimen was not modified during the study. None of the patients was on theophylline. Arterial blood gases were measured before and at 4, 8, and 12 hours after drug administration. Arterial oxygen saturation (SaO_2_) was measured continuously using a pulse oxymeter (Critikon, Tampa, FL, USA). All patients were ventilated in a semirecumbent position (which was kept constant throughout the study period), certain nursing interventions (for example, chest physiotherapy and suctioning) were withheld or minimized, and the remaining aspects of patient care (for example, fluids and nutrition) were at the discretion of the treating physician.

Respiratory system mechanics and heart rate (HR) were assessed before (baseline) and at 15, 30, 60 minutes as well as at 2, 3, 4, 6, 8, 10, and 12 hours after each series of puffs. The mechanical properties of the respiratory system were determined while the patient was ventilated at baseline ventilator settings shown in Table 1 using the occlusion technique, as previously described [25,26]. End-inspiratory static compliance of the respiratory system (Crs, st) as well as minimum (R_int_) and maximum (Rrs) resistance of the respiratory system were computed according to standard formulas [25,26]. The difference between Rrs and R_int _(ΔR), caused by time-constant inequalities and/or viscoelastic behavior (stress relaxation), was also calculated. R_int _and Rrs were corrected for the finite occlusion time of the occlusion valve of the ventilator [27]. The endotracheal tube resistance was not taken into account since each patient served as his or her own control. The heat and moisture exchanger (HME) of the ventilator circuit was removed before drug administration and reconnected after drug delivery, as previously suggested [1]. Similarly, the HME was removed prior to all respiratory mechanics measurements. As an index of duration of the resulting bronchodilation, time after salmeterol administration that R_int _remained less than 85% of its baseline value was measured.

Data concerning the overall effects of salmeterol on respiratory system mechanics and HR during the 12-hour study period were analyzed by nonparametric Wilcoxon signed rank test. Correlation of bronchodilator response to patients' baseline characteristics as well as their baseline respiratory system mechanics was tested by linear regression analysis by using Spearman's correlation coefficients. The changes of postinhalational parameters of respiratory system mechanics from baseline were given in absolute and percentage decrease values. All results are expressed as mean ± standard deviation. A *P *value of less than 0.05 was considered statistically significant. The Statistical Package for Social Sciences for Windows version 15.0 (SPSS Inc., Chicago, IL, USA) was used for data analysis.

## Results

**Figure 1:**
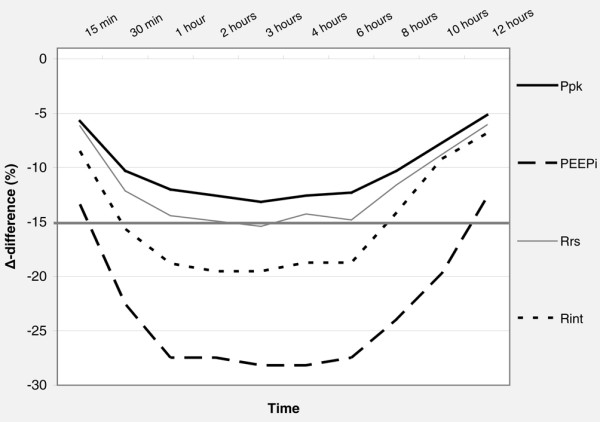
Percentage change (Δ-difference %) in peak airway pressure (Ppk), minimum inspiratory resistance (R_int_), maximum inspiratory resistance (Rrs), and intrinsic positive end-expiratory pressure (PEEPi) after salmeterol administration.

**Figure 2:**
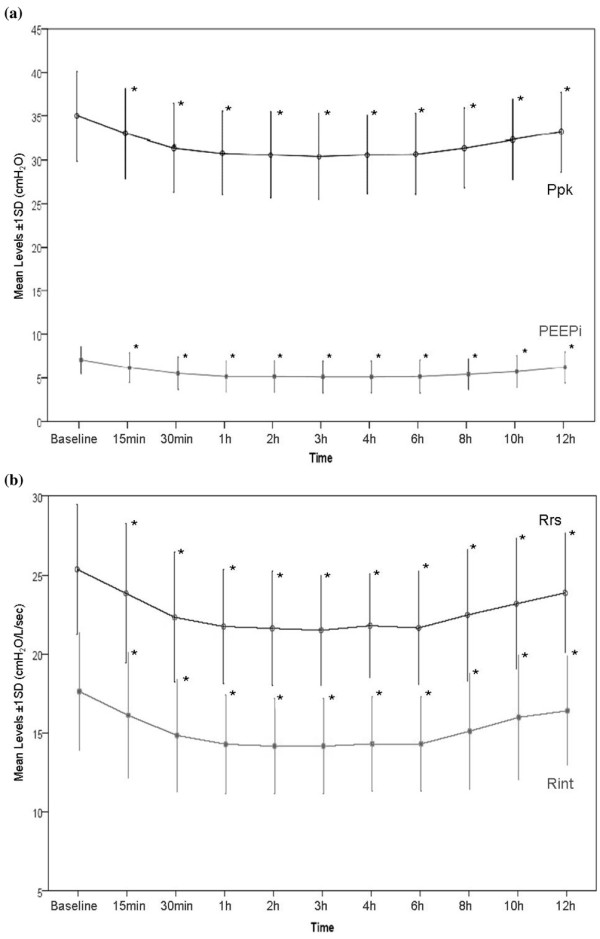
Effect of salmeterol on airway pressures and inspiratory resistance mean values. **(a) **Mean values (± 1 SD) of peak airway pressure (Ppk) and intrinsic positive end-expiratory pressure (PEEPi) at baseline and up to 12 hours after salmeterol administration. **(b) **Mean values (± 1 SD) of minimum inspiratory resistance (R_int_) and maximum inspiratory resistance (Rrs) at baseline and up to 12 hours after salmeterol administration. *Significantly different from baseline values (*P *< 0.05, Wilcoxon signed rank test). SD, standard deviation.

**Figure 3:**
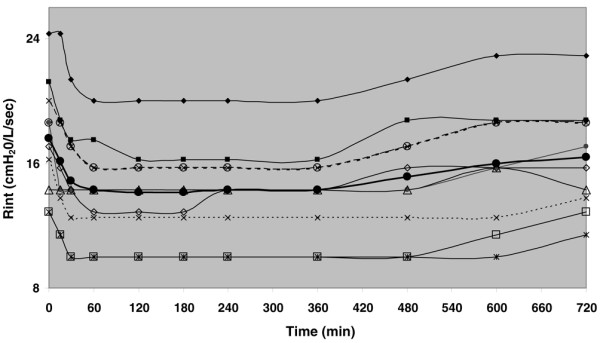
Individual patient values of minimum inspiratory resistance (R_int_) before and after salmeterol administration. Baseline values (before salmeterol) are indicated at time 0. The closed circles connected by the thick solid line represent the mean R_int _value for the whole patient group. -◆-: Patient #1; -■-: Patient #2; -x-: Patient #3; -✷-: Patient #4; -○-: Patient #5; -●-: Patient #6; -□-: Patient #7; -△: Patient #8; --x--: Patient #9; -◇-: Patient #10.

**Figure 4:**
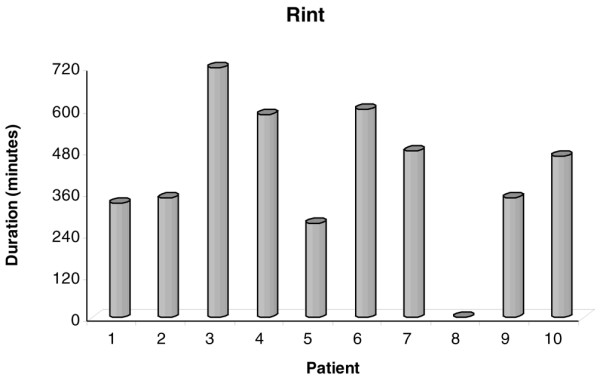
Time after salmeterol administration that minimum inspiratory resistance (R_int_) remained less than 85% of baseline in each patient.

Dynamic and static Paws, respiratory system mechanics, and HR before (time 0) and after salmeterol administration are shown in Table 2. The administration of 100 μg of salmeterol caused a significant decrease in dynamic and static Paws as well as in R_int_, Rrs, and intrinsic positive end-expiratory pressure (PEEPi) (Wilcoxon signed rank test). The decrease in Rrs was due mainly to a decrease in R_int _while ΔR remained relatively constant throughout the study period. As shown in Figures 1 and 2b, the effects of salmeterol on R_int_, which reflects 'ohmic' airway resistance, were evident at 30 minutes after drug delivery and remained relatively constant for approximately 8 hours. Except for one patient who did not exhibit any bronchodilator response to salmeterol, in the remaining nine patients mean R_int _changed from baseline by 17.8% ± 5.2% at 30 minutes, reaching a peak decrease of 21.6% ± 2.8% at 2 hours, while at 8 hours after drug administration a significant decrease of 16.2% ± 6.2% was still present (*P *< 0.05 for all values). Individual patient values of R_int _at baseline and at the predefined time points during the 12-hour study period after salmeterol administration are shown in Figure 3. Duration of bronchodilator response for each patient, expressed as the time that individual R_int _values remained less than 85% of their corresponding baseline values, is shown in Figure 4. Of note, in one patient, a sustained bronchodilator response was still present at 12 hours whereas four patients still exhibited a significant response at 8 hours after salmeterol administration. R_int _remained less than 85% of baseline values for 6 hours in three patients and for 4 hours in one patient, whereas one patient did not exhibit any bronchodilator response to salmeterol. Individual PEEPi and Rrs changes followed the R_int _response in a similar fashion.

**Table 2:** Airway pressures, respiratory system mechanics, and heart rate before and up to 12 hours after salmeterol administration

	Baseline	15 minutes	30 minutes	1 hour	2 hours	3 hours	4 hours	6 hours	8 hours	10 hours	12 hours
Ppk	35.0 ± 5.1	33.0 ± 5.1^a^	31.4 ± 5.0^a^	30.8 ± 4.8^a^	30.6 ± 4.9^a^	30.4 ± 4.9^a^	30.6 ± 4.5^a^	30.7 ± 4.6^a^	31.4 ± 4.5^a^	32.3 ± 4.5^a^	33.2 ± 4.5^a^

P_1_	22.3 ± 4.1	21.4 ± 4.3^a^	20.7 ± 4.4^a^	20.5 ± 4.2^a^	20.4 ± 4.4^a^	20.2 ± 4.1^a^	20.3 ± 3.8^a^	20.4 ± 3.9^a^	20.5 ± 3.6^a^	20.9 ± 3.7^a^	21.4 ± 3.7^a^

P_2_	16.7 ± 3.1	15.8 ± 2.8^a^	15.3 ± 3.1^a^	15.1 ± 2.9^a^	15.0 ± 3.1^a^	14.9 ± 3.2^a^	14.9 ± 2.8^a^	15.0 ± 2.8^a^	15.2 ± 2.7^a^	15.5 ± 2.6^a^	16.0 ± 2.7

PEEPi	7.1 ± 1.6	6.2 ± 1.7^a^	5.5 ± 1.9^a^	5.2 ± 1.8^a^	5.2 ± 1.8^a^	5.1 ± 1.8^a^	5.1 ± 1.8^a^	5.2 ± 1.9^a^	5.4 ± 1.8^a^	5.7 ± 1.8^a^	6.2 ± 1.8^a^

Rrs	25.4 ± 4.1	23.8 ± 4.4^a^	22.3 ± 4.1^a^	21.7 ± 3.6^a^	21.6 ± 3.6^a^	21.5 ± 3.5^a^	21.8 ± 3.3^a^	21.6 ± 3.6^a^	22.5 ± 4.2^a^	23.2 ± 4.1^a^	23.9 ± 3.8^a^

R_int_	17.6 ± 3.7	16.1 ± 4.0^a^	14.9 ± 3.6^a^	14.3 ± 3.1^a^	14.2 ± 3.0^a^	14.2 ± 3.0^a^	14.3 ± 3.0^a^	14.3 ± 3.0^a^	15.1 ± 3.7^a^	16.0 ± 3.9^a^	16.4 ± 3.4^a^

ΔR	7.7 ± 2.2	7.7 ± 2.5	7.5 ± 2.2	7.5 ± 2.2	7.5 ± 2.2	7.3 ± 1.9	7.5 ± 1.9	7.3 ± 2.0	7.3 ± 2.0	7.2 ± 2.0	7.5 ± 2.1

Cst, rs	54.3 ± 9.0	54.2 ± 9.0	53.1 ± 7.4	52.3 ± 7.4	53.0 ± 7.4	53.3 ± 7.5	53.1 ± 7.7	52.7 ± 6.7	52.8 ± 6.3	52.2 ± 5.8	52.2 ± 5.8

HR	74.8 ± 8.5	75.0 ± 7.3	74.5 ± 6.8	75.0 ± 7.2	74.5 ± 6.2	73.9 ± 7.7	73.5 ± 8.7	73.1 ± 9.6	73.8 ± 8.8	74.8 ± 11.0	73.4 ± 10.2

Values are presented as mean ± standard deviation. ^a^Significantly different from baseline values (*P *< 0.05, Wilcoxon signed rank test). ^b^Respiratory system mechanics were measured while the patients were on volume control with square wave inspiratory flow-time profile. Crs, st, end-inspiratory static compliance of the respiratory system (mL/cm H_2_O); HR, heart rate (beats per minute); P_1_, airway pressure at the point of zero flow (cm H_2_O); P_2_, plateau pressure (cm H_2_O); PEEPi, intrinsic positive end-expiratory pressure (cm H_2_O); Ppk, peak airway pressure (cm H_2_O); R_int_, minimum inspiratory resistance (cm H_2_O/L per second); Rrs, maximum inspiratory resistance (cm H_2_O/L per second); ΔR, difference between maximum inspiratory resistance and minimum inspiratory resistance (cm H_2_O/L per second).

Salmeterol administration had no substantial effect on gas exchange. Partial pressure of arterial oxygen (PaO_2_) differed from baseline by 0.9 ± 2.8, 1.3 ± 3.4, and 2.4 ± 4.9 mm Hg at 4, 8, and 12 hours, respectively (*P *> 0.05 for all values, Wilcoxon signed rank test), whereas a small (albeit statistically significant) decrease in PaCO_2 _(partial pressure of arterial carbon dioxide) was noted (-2.8 ± 1.3, -3.7 ± 1.7, and -4.1 ± 2 mm Hg at 4, 8, and 12 hours after salmeterol administration, respectively, *P *< 0.05 for all values, Wilcoxon signed rank test) as the observed bronchodilative effect resulted in improved alveolar ventilation. Changes in Cst, rs and HR were not significant at any time interval after salmeterol administration (Table 2). SaO_2 _remained constant throughout the study period, indicating that clinically significant changes in PaO_2 _as a result of salmeterol administration did not occur.

## Discussion

Our work demonstrates that 100 μg of the long-acting β2-agonist salmeterol delivered by MDI and a spacer caused a substantial bronchodilator effect that induced a significant and sustained decrease in inspiratory resistance indices, namely a maximal R_int _and Rrs decrease of 21.6% and 16.2%, respectively. These values compare rather well with the corresponding values in previous studies of short-acting β2-agonists in mechanically ventilated COPD patients, ranging between 18% and 25% for R_int _and between 8% and 15% for Rrs [11-17,28], while baseline respiratory mechanics in these studies were similar to ours (R_int _of 14 to 21 cm H_2_O/L per second and Rrs of 20 to 26 cm H_2_O/L per second). ΔR remained relatively constant throughout the study, indicating that salmeterol acted by dilating the central airways in a manner similar to salbutamol in previous studies [11-17]. Although expiratory resistance was not measured in the present study, this most probably was decreased by salmeterol, as indicated by the significant reduction in PEEPi and end-inspiratory static plateau pressure, which are indirect indices of dynamic hyperinflation. Similarly, although lung volumes were not assessed, the observed reduction in PEEPi could be regarded as an index – although an indirect one – of a corresponding change in end-expiratory lung volume (that is, a decrease in dynamic hyperinflation). We further showed that the onset of the bronchodilator response was noted at 30 minutes after drug delivery while its duration was quite variable among patients, remaining relatively constant for approximately 6 hours in the majority of our study population.

It is of interest that the assessment of bronchodilator response to β2-agonists in mechanically ventilated patients with COPD exacerbation has been limited mainly to the short-acting β2-agonist salbutamol. Although the bronchodilator effect of salmeterol has been studied in stable spontaneously breathing COPD patients [20-23] (where it has been shown to be effective for up to 12 hours, providing obvious advantages over short-acting therapies), no data exist concerning the effect of salmeterol in mechanically ventilated patients with COPD exacerbation. To the best of our knowledge, this is the first study about the effect of a long-acting bronchodilator on respiratory system mechanics and dynamic hyperinflation in mechanically ventilated COPD patients.

There are several limitations to our study. Since the optimal dose of inhaled β2-agonists in mechanically ventilated COPD patients has not been established clearly and based on the finding that aerosol deposition in mechanically ventilated patients is substantially lower than in nonintubated patients, higher doses have been recommended for ventilated patients [29]. Subsequent dose-response studies with salbutamol concluded that a dose of 400 μg ensures the best combination of bronchodilator effect and safety in mechanically ventilated patients with COPD [15,16]. In the case of salmeterol, however, due to the complete absence of relevant data, a dose of 100 μg was arbitrarily chosen by extrapolating the results of the salbutamol dose-response studies, in which the delivered dose was twice the dose recommended for nonintubated patients. This, however, does not exclude the possibility that, in an individual patient, higher doses may be necessary in order to achieve maximum bronchodilation. For example, it is not known whether patient 8 would have responded to a higher dose of salmeterol. Hence, in the absence of dose-response studies combined with appropriate pharmacokinetic data in regard to salmeterol administration in mechanically ventilated patients, straightforward interpretation of our results is a matter of discussion.

In nonintubated spontaneously breathing COPD patients, 50 μg of salmeterol causes a bronchodilator effect lasting at least 12 hours [20-23]. In the majority of our patients, the observed bronchodilator effect remained relatively constant for approximately 6 hours, indicating a reduced duration of the bronchodilator response during invasive mechanical ventilation. This could be explained by the reduced bioavailability of the drug, as observed by Duarte and colleagues [30] in the case of salbutamol. Furthermore, reduced duration of bronchodilator effect during COPD exacerbation has been observed for short-acting β2-agonists [31]. A decrease in pulmonary disposition and drug absorption in the setting of increased bronchomotor tone due to airway inflammation has been proposed as a possible underlying mechanism [32], an observation that could similarly apply to long-acting β2-agonists.

Propofol has been shown to cause a bronchodilative effect in mechanically ventilated COPD patients when administered as a bolus [33]. Although our patients were already receiving a continuous propofol infusion at a steady dose for at least 24 hours before study inclusion, the possibility of an additive to salmeterol bronchodilative effect cannot be entirely ruled out.

A considerable degree of variation in regard to the duration and magnitude of the bronchodilator response was observed. This response was, furthermore, rather unpredictable. Neither individual baseline characteristics or respiratory system mechanics nor the acute bronchodilator response (that is, at 30 minutes after salmeterol administration) was able to predict the duration of the resulting drug effect. The subject of interindividual variation in bronchodilator response to inhaled β2-agonists in COPD patients is a well-known entity. This variation seems to be determined by several factors, like age [34], the degree of baseline airflow limitation [35], and smoking status [36]. Furthermore, a potential role for genetic factors like polymorphisms of the β2-adrenergic receptor gene in determining bronchodilator response in COPD patients has recently been proposed [37]. Although these observations are limited to short-acting β2-agonists in non-intensive care unit patients and have to be regarded as preliminary evidence awaiting confirmation by larger studies, we believe their implications are nonetheless quite intriguing.

In our study, a 15% decrease of R_int _from baseline was defined as a significant bronchodilative response. Although there are no established threshold values, previous studies have shown that an Rrs or R_int _decrease of greater than 10% may indicate significant bronchodilator response [11-17,28]. Dhand and colleagues [15] examined R_int _variability in passively ventilated COPD patients over the course of 1 hour and found that the coefficient of variation among patients ranged from 1.6% to 3.9%. Although R_int _variability over the course of a 12-hour period is not known, it seems highly unlikely that a 15% decrease in R_int _would represent merely a patient's airway caliber variability rather than true bronchodilator response.

Therefore, according to our findings, it is not possible to propose a fixed dosing interval for salmeterol in mechanically ventilated patients with COPD exacerbation. Instead of the use of a standard dosing regimen, individualization of the dose interval titrated to objective indices of bronchodilation seems to be more prudent. The issue of objectively guided dose titration is probably the most important one for the clinician, whereas it certainly is a matter of debate which indices at which time point could serve as a reliable marker of effective bronchodilation. As shown in Figure 2a, we observed a decline in peak airway pressure (P_pk_) of approximately 4 cm H_2_O at 30 minutes after drug administration, a change that one could argue is of clinical relevance and relatively easy to detect at the bedside. However, further studies designed to specifically address these particular issues would certainly provide additional answers.

## Conclusion

Our study demonstrated that, in mechanically ventilated patients with COPD exacerbation, four puffs of salmeterol delivered by an MDI and a spacer device induced significant bronchodilation. The duration of the observed bronchodilator effect was highly variable and unpredictable among patients. This variability precludes definite guidelines in regard to an optimum dosing schedule, which should be titrated according to objective indices of bronchodilation.

## Key messages

• In mechanically ventilated patients with chronic obstructive pulmonary disease exacerbation, 100 μg of the long-acting β2-agonist salmeterol delivered by metered-dose inhaler and a spacer caused a significant bronchodilator effect.

• This effect was evident at 30 minutes after drug delivery and remained relatively constant for approximately 8 hours.

• Due to substantial interpatient variability, no definite conclusions in regard to the optimum dosing schedules of salmeterol can be drawn.

## Abbreviations

COPD: chronic obstructive pulmonary disease; Crs, st: end-inspiratory static compliance of the respiratory system; HME: heat and moisture exchanger; HR: heart rate; MDI: metered-dose inhaler; PaO_2_: partial pressure of arterial oxygen; Paw: airway pressure; PEEPi: intrinsic positive end-expiratory pressure; R_int_: minimum inspiratory resistance; Rrs: maximum inspiratory resistance; ΔR: difference between maximum inspiratory resistance and minimum inspiratory resistance; SaO_2_: arterial oxygen saturation; VT: tidal volume.

## Competing interests

The authors declare that they have no competing interests.

## Authors' contributions

DG conceived the study, participated in its design, and revised the final version of the manuscript for important intellectual content. PM participated in study design and data collection and drafted the manuscript. GG participated in data collection and helped to draft the manuscript. ML performed the statistical analysis and helped to draft the manuscript. All authors read and approved the final manuscript.
